# Low Platelet Count Predicts Reduced Survival in Potentially Resectable Hepatocellular Carcinoma

**DOI:** 10.3390/curroncol29030124

**Published:** 2022-02-28

**Authors:** Christopher Schrecker, Oliver Waidmann, Hanan El Youzouri, Jörg Trojan, Andreas Anton Schnitzbauer, Wolf Otto Bechstein, Stefan Zeuzem, Christine Koch

**Affiliations:** 1 Department of Medicine, University Hospital Frankfurt, Theodor-Stern-Kai 7, 60590 Frankfurt, Germany; oliver.waidmann@kgu.de (O.W.); trojan@em.uni-frankfurt.de (J.T.); stefan.zeuzem@kgu.de (S.Z.); 2 Department of Surgery, University Hospital Frankfurt, Theodor-Stern-Kai 7, 60590 Frankfurt, Germany; hanan.elyouzouri@kgu.de (H.E.Y.); andreasanton.schnitzbauer@kgu.de (A.A.S.); wolf.bechstein@kgu.de (W.O.B.)

**Keywords:** hepatocellular carcinoma, platelet count, thrombocytopenia, thrombocytosis, cirrhosis, portal hypertension, overall survival, perioperative mortality

## Abstract

The prognostic role of platelet count in hepatocellular carcinoma (HCC) remains unclear, and in fact both thrombocytopenia and thrombocytosis are reported as predictors of unfavourable outcomes. This study aimed to clarify the prognostic value of preoperative platelet count in potentially resectable HCC. We retrospectively reviewed 128 patients who underwent hepatic resection for HCC at a tertiary academic centre (2007–2019). Patient data were modelled by regression analysis, and platelet count was treated as a continuous variable. 89 patients had BCLC 0/A tumours and 39 had BCLC B tumours. Platelet count was higher in patients with larger tumours and lower in patients with higher MELD scores, advanced fibrosis, and portal hypertension (*p* < 0.001 for all listed variables). After adjusting for BCLC stage and tumour diameter, low platelet count associated with reduced overall survival (hazard ratio 1.25 per 50/nL decrease in platelet count, 95% confidence interval (CI) 1.02–1.53, *p* = 0.034) and increased perioperative mortality (odds ratio 1.96 per 50/nL decrease in platelet count, 95% CI 1.19–3.53, *p* = 0.014). Overall, low platelet count correlates with increased liver disease severity, inferior survival, and excess perioperative mortality in resectable HCC. These insights might be applied in clinical practice to better select patients for resection.

## 1. Introduction

Hepatocellular carcinoma (HCC) is a leading cause of cancer-related morbidity and mortality [1], and typically arises in cirrhotic livers with underlying viral or alcoholic liver disease [2]. Patients may present with acute decompensation of their underlying liver disease, or through routine screening of at-risk populations by ultrasound scanning and measurement of alpha-fetoprotein (AFP) levels [3]. A suspected diagnosis of HCC is then confirmed by contrast-enhanced imaging and liver biopsy in patients who are suitable for treatment. Therapy is guided by the Barcelona Clinic Liver Cancer (BCLC) staging system, which takes into account performance status, severity of the underlying liver disease, tumour size, and spread [4]. Treatment for early stage tumours is with curative intent, and options include hepatic resection, liver transplantation, and minimally invasive ablation therapies [5,6]. More advanced tumours are treated by chemoembolisation and systemic therapies, but in advanced disease, patient outcomes remain exceedingly poor even with treatment [5,6].

Platelet count has drawn much attention as a potential biomarker in patients with HCC. This is unsurprising, given the routine nature of this laboratory test and the well-established interplay between platelet and tumour biology [7]. Indeed, elevated platelet counts and paraneoplastic thrombotic events are hallmarks of many solid tumours, including HCC [8,9], and appear to be driven by tumour-derived procoagulant factors and inflammatory cytokines [10]. Beyond their involvement in paraneoplastic phenomena, platelets can be hijacked by tumour cells to actively enhance tumour growth and metastatic potential [11]. For instance, platelets may dampen antitumour immunity [12], release tumour-promoting cargo into the tumour microenvironment [13], and aggregate around circulating tumour cells to shield them from immune surveillance [14]. Supporting the notion that platelets can fuel cancer aggressiveness, long-term antiplatelet therapy has been shown to prevent colon cancer in patients with Lynch syndrome [15] and reduce incident HCC in patients with viral hepatitis [16]. It follows that platelet count has great potential as a biomarker of malignant disease, including HCC.

However, despite its expected utility as a measure of cancer aggressiveness and patient outcomes, the informational value of platelet count in patients with HCC remains unclear. This is because hepatocellular tumours may well elicit a paraneoplastic thrombocytosis, but the underlying liver disease is often accompanied by a drop in platelet numbers owing to increased pooling of platelets in an enlarged spleen (a phenomenon known as hypersplenism). Hypersplenism, in turn, is a direct consequence of hepatic architectural changes and portal hypertension in patients with chronic liver disease [17]. In addition to platelet sequestration in the splenic sinusoids, reduced hepatic output of thrombopoietin in the setting of synthetic liver failure puts a brake on megakaryopoiesis and platelet production [18]. Thus, hepatocellular tumours are unique among the solid malignancies in that some may be accompanied by thrombocytopenia, and others by thrombocytosis. The underlying liver disease therefore adds a layer of complexity to the interpretation of platelet counts in HCC.

In the present study, we aimed to clarify the prognostic value of preoperative platelet count in the setting of resectable HCC. Despite the breadth of previous work on platelet kinetics in HCC, there remains a lack of clarity over the prognostic role of platelet count; in fact, both thrombocytopenia and thrombocytosis are reported as predictors of unfavourable patient outcomes [19]. Herein, we studied a cohort of 128 patients undergoing hepatic resection for HCC, using statistical regression models to explore the association of platelet count with survival outcomes and perioperative mortality, whilst systematically adjusting for measures of HCC tumour burden.

## 2. Materials and Methods

### 2.1. Study Design

**Figure 1:**
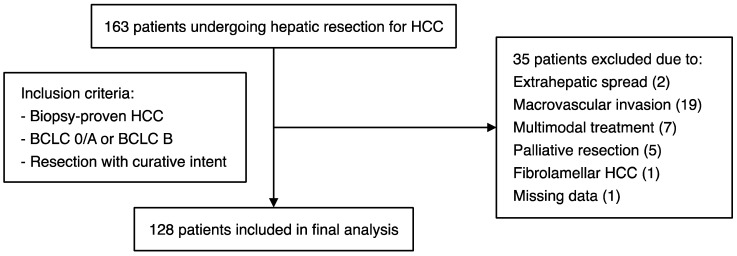
Schematic representation of the patient cohort, with details of the inclusion/exclusion criteria applied for this study.

We retrospectively analysed the clinical and laboratory data of patients who underwent hepatic resection for HCC at the University Hospital Frankfurt, a German referral centre for liver diseases (February 2007–May 2019). Patients were included in the study if they had biopsy-proven HCC (BCLC stage 0/A or B) and if they were operated on with curative intent (Figure 1). Surgery was recommended by a multidisciplinary tumour board at the University Hospital Frankfurt. Patients were excluded if they had advanced (BCLC C) disease with macrovascular invasion or extrahepatic spread, if they were operated on with palliative intent, or if histology revealed an alternative diagnosis such as intrahepatic cholangiocarcinoma or fibrolamellar HCC (Figure 1). The study was approved by the institutional review board of the University Hospital Frankfurt (reference number SGI-4-2019).

### 2.2. Definitions

Table 1 provides a summary of the patient database created for this study. All patient data, including laboratory parameters, pathology reports, staging and prognostic scores, and survival outcomes, were manually verified for accuracy and completeness. Platelet counts and other relevant laboratory parameters were measured in the immediate run-up to surgery. BCLC stage was determined based on preoperative imaging and pathological assessment of the resectate, and maximum tumour diameter was defined as the maximum diameter of the largest HCC nodule. Solitary tumours without macrovascular invasion or extrahepatic spread were classified as BCLC A irrespective of their size [5]. BCLC stage was used as a measure of tumour burden for the purpose of this study, because BCLC stages 0/A and B differ only in terms of the size and number of HCC nodules (not performance status or hepatic function) [4]. The Milan and Up-to-Seven criteria were applied as previously described [20,21]. The Child–Pugh score was used to estimate hepatic function in all patients of our cohort and was calculated as per the original publication [22]. Although the Child–Pugh system was originally developed to predict survival outcomes in patients with cirrhosis, it is currently used to assess hepatic function in all patients with HCC, both with and without underlying cirrhosis (e.g., in the RESORCE and REFLECT trials [23,24]). Model for end-stage liver disease (MELD) and albumin–bilirubin (ALBI) scores were calculated as follows: MELD = 9.57 × ln(creatinine) + 3.78 × ln(bilirubin) + 11.2 × ln(INR) + 6.43 (creatinine and bilirubin expressed in milligrams per decilitre (mg/dL)) [25]; ALBI = log_10_(bilirubin) × 0.66 + albumin × −0.085 (bilirubin expressed in micromoles per litre (μmol/L), albumin expressed in grams per litre (g/L)) [26]. The histologic degree of hepatic fibrosis was graded according to the METAVIR scoring system (F3/F4 for presence of advanced fibrosis/cirrhosis vs. F0–F2 for absence of advanced fibrosis) [27]. Portal hypertension was diagnosed in patients with chronic liver disease and endoscopic evidence of oesophageal varices and/or portal hypertensive gastropathy [28]. Invasive portal haemodynamic studies are not routinely performed in patients with cirrhosis treated at our institution. Portal hypertension was not diagnosed based on the presence of ascites alone, given that transudative ascites resulting from portal hypertension cannot be reliably distinguished from exudative malignant ascites in patients with HCC.

### 2.3. Statistical Analysis

Data are expressed as the mean ± SEM or median and range, as appropriate. Platelet count was treated as a continuous variable unless otherwise stated. Statistical analysis was carried out in the R environment for statistical computing [29]. Patient survival was assessed from the time of hepatic resection until data closure on 31 January 2020, and the six patients who underwent liver transplantation following resection were censored upon transplantation. Survival data were modelled by Cox regression using the R survival package [30]. Linear/logistic regression models were constructed using the generalised linear model (glm) function in the standard R environment. Multivariable models were used to adjust for selected measures of HCC tumour burden (BCLC stage and maximum tumour diameter). Population means were compared by unpaired *t*-test (*t*.test function), and Pearson’s correlation coefficient was computed using the cor.test function. Results with a *p*-value < 0.05 were considered statistically significant.

## 3. Results

**Figure A1:**
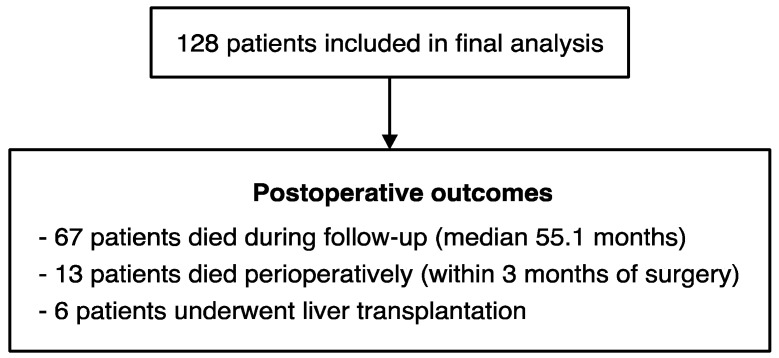
Schematic detailing the postoperative outcomes of the 128 patients included in the study.

The aim of this study was to clarify the prognostic value of preoperative platelet count in resectable HCC. We included 128 patients with HCC in the study, all of whom underwent hepatic resection with curative intent. Of these, 89 patients had BCLC 0/A tumours and 39 had BCLC B tumours. All participants had good performance status (ECOG 0/1) and adequate hepatic function (Child–Pugh A disease in 126 patients, Child–Pugh B7 disease in 2 patients). Patients were followed up for a median of 55.1 months (95% confidence interval (CI) 39.2–66.7), and a total of 67 deaths from all causes were recorded during the observation period. The median overall survival was 51.1 months (95% CI 33.0–71.9) in the full cohort, 53.7 months in BCLC 0/A patients, and 27.5 months in BCLC B patients. Baseline characteristics are summarised in Table 1, and postoperative outcomes are detailed in Figure A1.

### 3.1. Platelet Count Correlates with Tumour Burden and Liver Disease Severity in Resectable HCC

**Figure 2:**
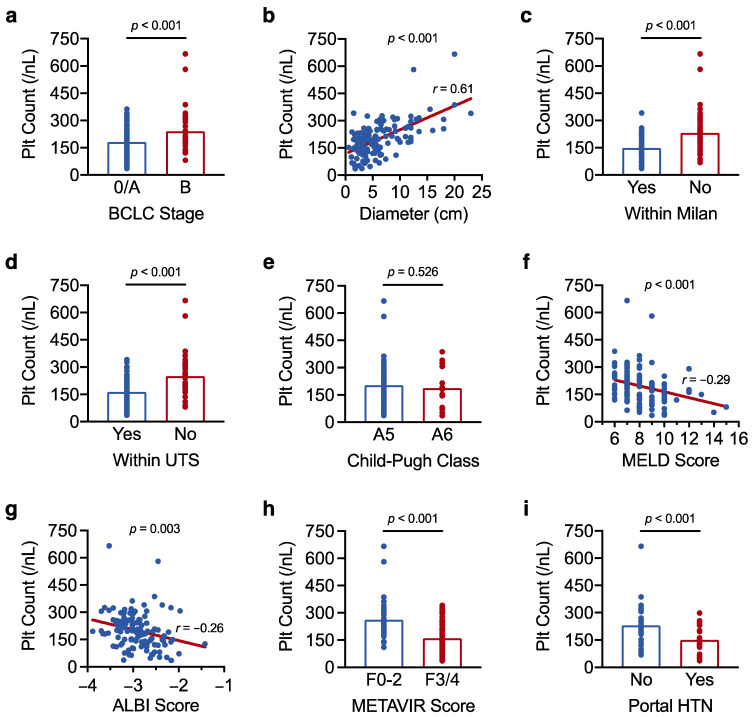
Platelet count correlates with tumour burden and liver disease severity in resectable HCC. (**a**–**i**) Results of a linear regression analysis showing the association of platelet count with (**a**) BCLC stage, (**b**) maximum tumour diameter, (**c**) the Milan criteria, (**d**) the Up-to-Seven (UTS) criteria, (**e**) Child–Pugh class, (**f**) MELD score, (**g**) ALBI score, (**h**) METAVIR fibrosis score, and (**i**) portal hypertension (HTN). Correlation coefficients (*r*) were determined by Pearson’s correlation analysis. The analysis was carried out in the full cohort of 128 patients in (**a**–**h**) and was limited to the 70 patients who underwent upper gastrointestinal endoscopy in (**i**). Results with a *p*-value < 0.05 were considered statistically significant.

HCC tumour burden and liver disease severity are major determinants of HCC prognosis [4]. To better understand the prognostic role of platelet count in resectable HCC, we therefore first examined the correlation of platelet count with selected measures of tumour burden (BCLC stage, maximum tumour diameter, Milan criteria, Up-to-Seven criteria) and liver disease severity (Child–Pugh class, MELD score, ALBI score, METAVIR fibrosis score, portal hypertension). Overall, we found that higher platelet count associated with greater tumour burden, and lower platelet count associated with more advanced liver disease. Specifically, platelet count was higher with increasing BCLC stage and tumour diameter, and in HCCs beyond the Milan and Up-to-Seven criteria, whereas platelet count was lower in patients with higher MELD, ALBI, and METAVIR scores, and in individuals with portal hypertension (Table 2 and Figure 2).

Taken together, we show that platelet count correlates with tumour burden and liver disease severity in resectable HCC. Importantly, our findings indicate that tumour burden and liver disease severity exert opposing effects on overall platelet count. As such, the paraneoplastic increase in platelet count that accompanies larger HCCs could mask the pronounced thrombocytopenia usually observed in patients with advanced liver disease and portal hypertension.

### 3.2. Low Platelet Count Associates with Reduced Overall Survival in Resectable HCC

**Figure 3:**
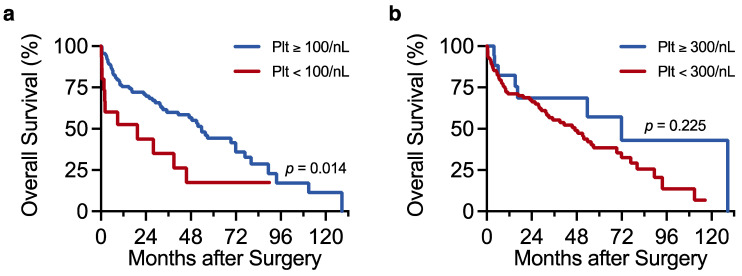
Low platelet count associates with reduced overall survival in resectable HCC. (**a**,**b**) Kaplan–Meier plots showing the association of platelet count (Plt) with overall survival. Overall survival was compared between patients with platelet counts ≥100/nL (*n* = 113) and <100/nL (*n* = 15) in (**a**) and between those with counts ≥300/nL (*n* = 17) and <300/nL (*n* = 111) in (**b**). Survival data were modelled by Cox regression analysis. Results with a *p*-value < 0.05 were considered statistically significant.

We next explored the association of platelet count with overall survival by Cox regression analysis. Since we demonstrated that platelet count correlates with established HCC prognostic factors (e.g., BCLC stage, ALBI score, portal hypertension), we hypothesised that platelet count may itself carry prognostic information. Platelet count was first analysed as a binary variable. Indeed, the 15 patients with platelet counts below 100/nL had particularly poor survival outcomes compared with the rest of the cohort (median overall survival 19.4 vs. 53.7 months, hazard ratio (HR) 2.26 for platelet counts <100/nL, 95% CI 1.18–4.34, *p* = 0.014) (Table 3 and Figure 3a). The prognostic power of low platelet count was retained after adjusting the Cox model for BCLC stage and tumour diameter (HR 3.20 for platelet counts <100/nL, 95% CI 1.58–6.48, *p* = 0.001) (Table A1). By contrast, overall survival was no different in the 17 patients with the highest platelet counts (≥300/nL) compared with the rest of the cohort (*p* = 0.225) (Figure 3b). The prognostic value of low platelet count may relate to its close association with increased liver disease severity and portal hypertension, both of which are established negative prognostic factors in HCC. Accordingly, our Cox analysis confirmed high MELD/ALBI scores and portal hypertension as predictors of reduced patient survival (Table 3).

When treated as a continuous variable, platelet count did not correlate with overall survival in univariate Cox analysis (*p* = 0.913) (Table 3). We reasoned that the prognostic information typically carried by low platelet count in advanced liver disease may have been distorted by the paraneoplastic increase in platelet count associated with greater HCC tumour burden. We therefore adjusted the univariate Cox model for BCLC stage and maximum tumour diameter to account for individual differences in tumour burden. Strikingly, low platelet count became a strong predictor of reduced survival in the multivariable model, after adjusting for BCLC stage and tumour diameter (HR 1.25 per 50/nL decrease in platelet count, 95% CI 1.02–1.53, *p* = 0.034) (Table A2). Our findings support the idea that low platelet count predicts inferior survival in resectable HCC, and introduce the notion that this prognostic information may be lost when individual tumour burden is not taken into account.

### 3.3. Low Platelet Count Increases the Risk of Perioperative Mortality in Resectable HCC

**Figure 4:**
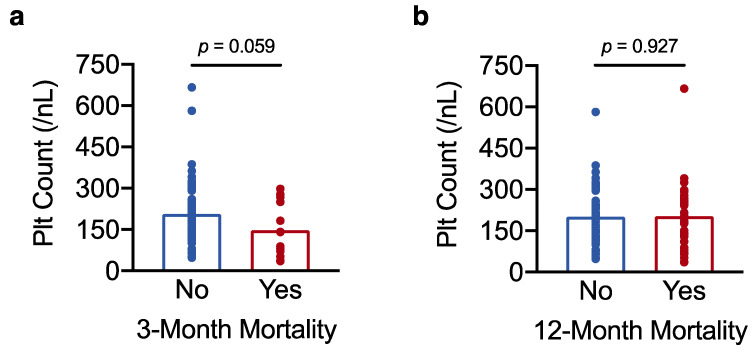
Low platelet count increases the risk of perioperative mortality in resectable HCC. (**a**,**b**) Results of a *t*-test analysis showing the association of platelet count with perioperative mortality (3-month postoperative mortality) in (**a**) and with 12-month postoperative mortality in (**b**). The result in (**a**) with a *p*-value < 0.1 was considered a statistical trend.

To further investigate why low platelet count associated with unfavourable survival outcomes, we then asked whether low platelet count also correlated with perioperative mortality. The question was addressed by logistic regression analysis with platelet count as the predictor variable and perioperative mortality as the outcome variable. Perioperative mortality was defined as mortality within 3 months after surgery [31]. In univariate analysis, low platelet count associated with increased perioperative mortality, such that for every 50/nL decrease in platelet count, the odds of death vs. survival at 3 months increased by more than 50% (odds ratio (OR) 1.55 for death vs. survival at 3 months, 95% CI 1.06–2.40, *p* = 0.034). Accordingly, platelet counts were lower in the 13 patients who died perioperatively (148 ± 27/nL) compared with the rest of the cohort (206 ± 9/nL) (Figure 4a). Low platelet count remained a predictor of perioperative death after adjusting the regression model for BCLC stage and maximum tumour diameter (OR 1.96 per 50/nL decrease in platelet count, 95% CI 1.19–3.53, *p* = 0.014). By contrast, platelet count no longer affected the odds of death vs. survival at 12 months post resection (*p* = 0.917), and platelet counts did not differ between patients who died within 12 months of surgery (202 ± 21/nL) and the rest of the cohort (200 ± 10/nL) (Figure 4b).

Taken together, this shows that lower platelet counts, which appear to reflect a higher degree of hepatic dysfunction/portal hypertension (Results, Section 3.1.) and surgical risk, increase the odds of perioperative mortality (but not 12-month postoperative mortality). The link between low preoperative platelet count and increased perioperative mortality may explain, at least in part, why low platelet count predicts reduced overall survival in resectable HCC.

### 3.4. Platelet Counts in the Normal Range Do Not Rule out Portal Hypertension in Resectable HCC

**Figure 5:**
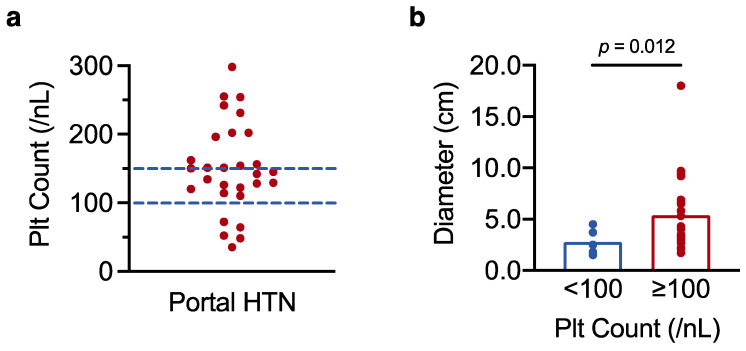
Platelet counts in the normal range do not rule out portal hypertension in resectable HCC. (**a**) Scatter plot showing individual platelet counts in the 29 patients with confirmed portal hypertension (HTN). (**b**) *t*-test analysis of the 29 patients with portal hypertension, comparing maximum tumour diameter in patients with platelet counts <100/nL and ≥100/nL. Results with a *p*-value < 0.05 were considered statistically significant.

Given the observed link between low platelet count and perioperative mortality, we finally asked whether preoperative platelet count could be used to identify patients with portal hypertension (an established measure of increased surgical risk [22]) who may be better served by locoregional therapies. HCC practice guidelines recommend a platelet cut-off score of 100/nL to rule in (<100/nL) or rule out (≥100/nL) the possibility of portal hypertension [5,6]. We therefore tested whether this cut-off score reliably discriminates between HCC patients with and without portal hypertension. Of the 70 patients for whom endoscopy data were available, oesophageal varices and/or portal hypertensive gastropathy were recorded in 29 patients. Among the 29 patients with manifest portal hypertension, only 5 had platelet counts below 100/nL, and almost half had platelet counts in the normal range (≥150/nL) (Figure 5a). In line with our results in Section 3.1., the 5 patients with portal hypertension and very low platelet counts had smaller tumours than the 24 patients with portal hypertension and higher platelet numbers (mean tumour size 2.8 ± 0.6 cm in patients with platelet counts <100/nL, vs. 5.4 ± 0.8 cm in patients with platelet counts ≥100/nL, *p* = 0.012 as determined by *t*-test analysis) (Figure 5b). Thus, platelet counts in the normal range do not exclude portal hypertension in patients with resectable HCC, particularly in individuals with greater tumour burden (which may be accompanied by a paraneoplastic increase in platelet count).

## 4. Discussion

In this study, we have shown that low preoperative platelet count predicts inferior survival outcomes in patients undergoing hepatic resection for HCC. We found that platelet count tends to be higher in patients with greater tumour burden and lower in patients with advanced fibrosis and portal hypertension. Our data indicate that the link between low platelet count and poor survival may result from the increased perioperative mortality that accompanies cirrhotic liver disease with portal hypertension, which is itself associated with thrombocytopenia. The fact that platelet count was analysed as a continuous variable implies that the observed relationship between lower platelet count and reduced survival applies across a wide range of platelet counts and is not limited to patients with particularly low platelet numbers.

Despite the ever-growing literature on platelet kinetics in HCC, the prognostic value of platelet count in HCC remains unclear. Some reports, including a meta-analysis of 33 studies [32], found that pre-treatment thrombocytopenia was a predictor of poor prognosis. Other studies have reported the exact opposite, namely, that it is patients with pre-treatment thrombocytosis who have particularly unfavourable outcomes [33]. Our linear regression model links high platelet count to increased tumour burden and low platelet count to more advanced liver disease, demonstrating that both thrombocytosis and thrombocytopenia may reflect disease processes that confer a negative prognosis. Thus, our findings illustrate how the seemingly contradictory results from previous studies can be reconciled. The abovementioned meta-analysis concluded that the small number of studies that measured platelet count as a continuous variable did not find an association between pre-treatment platelet count and survival outcomes [32]. The authors of the meta-analysis considered whether an existing association might have been overlooked because individual studies did not adjust for relevant confounders. Our data confirm that low pre-treatment platelet count does indeed associate with poor survival in resectable HCC, even when platelet count is treated as a continuous variable, but only after adjusting for tumour burden to account for the paraneoplastic increase in platelet count that accompanies larger tumours.

Notably, our findings apply primarily to the subset of patients with early stage HCC, undergoing hepatic resection with curative intent. In this context, lower platelet count appears to associate with inferior survival outcomes. As outlined above, this may relate to the link between thrombocytopenia and end-stage liver disease, which is itself a major driver of mortality in patients with HCC and cirrhosis. Another plausible explanation is that preoperative thrombocytopenia may associate with surgical risk and perioperative mortality, as indicated by our analysis. In line with our findings, previous reports of patients undergoing hepatic resection for HCC have linked low preoperative platelet count to higher rates of major surgical complications, postoperative liver insufficiency, and increased perioperative mortality [34,35]. That said, the association between platelet numbers and patient outcomes may be entirely different in advanced HCC, where patients with low platelet count appear to have superior survival outcomes [36]. A possible explanation is that, as hepatocellular tumours progress, tumour burden (linked to thrombocytosis) takes over from hepatic dysfunction (linked to thrombocytopenia) as the limiting factor in patient survival.

Importantly, we demonstrate that a nuanced understanding of platelet kinetics can avoid overlooking patients with occult portal hypertension (and increased perioperative risk) who may be better served by locoregional therapies than surgical resection. Clinical practice guidelines on the management of HCC recommend surgery in patients with solitary tumours, compensated liver disease, and a normal hepatic venous pressure gradient. According to the EASL and AASLD guidelines, platelet counts ≥100/nL indicate normal portal pressures [5,6]. Similarly, the Baveno criteria state that portal hypertension can be excluded in patients with chronic liver disease and platelet counts ≥150/nL (when liver stiffness measurements by transient elastography remain ≤15 kPa) [37]. In line with these recommendations, there are data suggesting that portal pressure correlates with platelet count in patients with cirrhosis [38,39]. In the setting of HCC, however, the anticipated correlation between portal pressure and platelet count is unreliable, as demonstrated by our analysis. Indeed, we showed that platelet counts in the normal range by no means rule out the possibility of portal hypertension in patients with HCC and cirrhosis. This may again be due to the paraneoplastic increase in platelet count that accompanies larger HCCs. We therefore strongly caution against the use of a platelet cut-off score to rule out portal hypertension in resectable HCC. Instead, we urge clinicians to screen their patients for signs of increased portal pressure and to incorporate a diagnostic upper endoscopy as a routine component of the preoperative workup.

## 5. Conclusions

Overall, this study clarifies the prognostic value of platelet count in resectable HCC and suggests how this knowledge could be applied in clinical practice. Although it is among the most routine of laboratory parameters, the correct interpretation of platelet count in the setting of HCC is far from trivial. We anticipate that our study will fill some of this knowledge gap and, in doing so, will positively impact on clinical decision making and patient outcomes.

## Figures and Tables

**Table 1:** Baseline characteristics.

Baseline Variable		Summary Statistics
Age [years]—median (range)		65 (34–81)
Gender—no. (%)	Male Female	96 (75.0) 32 (25.0)
Liver Disease Aetiology—no. (%)	HBV/HCV ASH/NASH Other	56 (43.8) 53 (41.4) 19 (14.8)
BCLC Stage—no. (%)	0/A B	89 (69.5) 39 (30.5)
Max. Diameter [cm]—median (range)		5.0 (0.6–23.0)
Number of Nodules—no. (%)	Single Two/three Multiple	87 (68.0) 19 (14.8) 22 (17.2)
Child–Pugh Class—no. (%)	A5 A6 B7	109 (85.2) 17 (13.3) 2 (1.6)
MELD Score—median (range)		7 (6–15)
ALBI Score—median (range)		−2.94 (−3.89 to −1.42)
METAVIR Score—no. (%)	F0–2 F3/4	50 (39.1) 78 (60.9)
Ascites—no. (%)	None Mild/moderate	117 (91.4) 11 (8.6)
Portal Hypertension—no. (%)		29/70 * (41.4)

Observations: The full cohort comprised 128 patients who underwent hepatic resection for HCC. Abbreviations: ALBI, albumin–bilirubin; ASH/NASH, alcoholic/non-alcoholic steatohepatitis; BCLC, Barcelona Clinic Liver Cancer; HBV, hepatitis B virus; HCV, hepatitis C virus; MELD, model for end-stage liver disease; METAVIR, a histologic fibrosis score. * Endoscopic signs of portal hypertension were recorded in 29 out of the 70 patients who received an upper gastrointestinal endoscopy within one year of hepatic resection.

**Table 2:** Prediction of platelet count by simple linear regression.

Predictor Variable	*β*-Coefficient * (± SE)	*p*-Value
BCLC B (vs. BCLC 0/A)	+59.6 (± 17.5)	<0.001
Max. Tumour Diameter (per 1 cm increase)	+13.2 (± 1.5)	<0.001
Milan Criteria (beyond vs. within)	+83.6 (± 15.6)	<0.001
Up-to-Seven Criteria (beyond vs. within)	+87.8 (± 14.9)	<0.001
Child–Pugh Class (A6 vs. A5)	−15.8 (± 24.9)	0.526
MELD Score (per unit increase)	−16.3 (± 4.7)	<0.001
ALBI Score (per unit increase)	−60.0 (± 19.6)	0.003
METAVIR Score (F3/4 vs. F0–2)	−102.3 (± 14.7)	<0.001
Portal HTN ** (presence vs. absence)	−79.5 (± 21.7)	<0.001

Observations: The full cohort comprised 128 patients who underwent hepatic resection for HCC. Abbreviations: ALBI, albumin–bilirubin; BCLC, Barcelona Clinic Liver Cancer; HTN, hypertension; MELD, model for end-stage liver disease; METAVIR, a histologic fibrosis score; SE, standard error. * *β*-Coefficient: degree of change in the outcome variable (platelet count) for every unit of change in the predictor variable. ** Analysis limited to the 70 patients who underwent upper GI endoscopy.

**Table 3:** Analysis of overall survival by simple Cox regression.

Predictor Variable	HR (95% CI)	*p*-Value
Patient Age (per unit increase)	1.00 (0.98–1.03)	0.775
Thrombocytopenia (Plt Count < 100/nL) *	2.26 (1.18–4.34)	0.014
Platelet Count (per 50/nL decrease) **	1.01 (0.88–1.16)	0.913
BCLC B (vs. BCLC 0/A)	1.30 (0.78–2.18)	0.314
Max. Diameter (per 1 cm increase)	1.05 (1.00–1.10)	0.040
Child–Pugh Class (A6 vs. A5)	2.04 (1.03–4.03)	0.041
MELD Score (per unit increase)	1.29 (1.11–1.49)	<0.001
ALBI Score (per unit increase)	1.92 (1.09–3.39)	0.025
METAVIR Score (F3/4 vs. F0–2)	0.97 (0.59–1.59)	0.905
Portal HTN *** (presence vs. absence)	3.64 (1.69–7.84)	<0.001

Observations: 128 patients who underwent hepatic resection for HCC; Number of events: 67 deaths. Abbreviations: ALBI, albumin-bilirubin; BCLC, Barcelona Clinic Liver Cancer; CI, confidence interval; HR, hazard ratio; HTN, hypertension; MELD, model for end-stage liver disease; METAVIR, a histologic fibrosis score. */** Platelet count analysed as a binary (*) or as a continuous (**) variable. *** Analysis limited to the 70 patients who underwent upper GI endoscopy.

## Data Availability

The anonymised patient database is available upon request.

## Appendix A

Figure A1: Schematic detailing the postoperative outcomes of the 128 patients included in the study; Table A1: Overall survival analysed by multiple Cox regression (plt count: binary variable); Table A2: Overall survival analysed by multiple Cox regression (plt count: continuous variable).

**Table A1:** Overall survival analysed by multiple Cox regression (plt count: binary variable).

Predictor Variable	HR (95% CI)	*p*-Value
Thrombocytopenia (Plt Count < 100/nL)	3.20 (1.58–6.48)	0.001
BCLC B (vs. BCLC 0/A)	1.30 (0.76–2.21)	0.340
Max. Diameter (per 1 cm increase)	1.07 (1.02–1.13)	0.010

Observations: 128 patients who underwent hepatic resection for HCC; Number of events: 67 deaths. Abbreviations: BCLC, Barcelona Clinic Liver Cancer; CI, confidence interval; HR, hazard ratio.

**Table A2:** Overall survival analysed by multiple Cox regression (plt count: continuous variable).

Predictor Variable	HR (95% CI)	*p*-Value
Platelet Count (per 50/nL decrease)	1.25 (1.02–1.53)	0.034
BCLC B (vs. BCLC 0/A)	1.47 (0.85–2.57)	0.172
Max. Diameter (per 1 cm increase)	1.11 (1.03–1.19)	0.004

Observations: 128 patients who underwent hepatic resection for HCC; Number of events: 67 deaths. Abbreviations: BCLC, Barcelona Clinic Liver Cancer; CI, confidence interval; HR, hazard ratio.

## References

[1] Bray F., Ferlay J., Soerjomataram I., Siegel R.L., Torre L.A., Jemal A. (2018). Global cancer statistics 2018: GLOBOCAN estimates of incidence and mortality worldwide for 36 cancers in 185 countries. CA Cancer J. Clin..

[2] Villanueva A. (2019). Hepatocellular Carcinoma. N. Engl. J. Med..

[3] Tzartzeva K., Obi J., Rich N.E., Parikh N.D., Marrero J.A., Yopp A., Waljee A.K., Singal A.G. (2018). Surveillance Imaging and Alpha Fetoprotein for Early Detection of Hepatocellular Carcinoma in Patients With Cirrhosis: A Meta-analysis. Gastroenterology.

[4] Llovet J.M., Brú C., Bruix J. (1999). Prognosis of hepatocellular carcinoma: The BCLC staging classification. Semin. Liver Dis..

[5] Galle P.R., Forner A., Llovet J.M., Mazzaferro V., Piscaglia F., Raoul J.L., Schirmacher P., Vilgrain V. (2018). EASL Clinical Practice Guidelines: Management of hepatocellular carcinoma. J. Hepatol..

[6] Heimbach J.K., Kulik L.M., Finn R.S., Sirlin C.B., Abecassis M.M., Roberts L.R., Zhu A.X., Murad M.H., Marrero J.A. (2018). AASLD guidelines for the treatment of hepatocellular carcinoma. Hepatology.

[7] Gaertner F., Massberg S. (2019). Patrolling the vascular borders: Platelets in immunity to infection and cancer. Nat. Rev. Immunol..

[8] Haemmerle M., Stone R.L., Menter D.G., Afshar-Kharghan V., Sood A.K. (2018). The Platelet Lifeline to Cancer: Challenges and Opportunities. Cancer Cell.

[9] Varki A. (2007). Trousseau’s syndrome: Multiple definitions and multiple mechanisms. Blood.

[10] Stone R.L., Nick A.M., McNeish I.A., Balkwill F., Han H.D., Bottsford-Miller J., Rupairmoole R., Armaiz-Pena G.N., Pecot C.V., Coward J. (2012). Paraneoplastic thrombocytosis in ovarian cancer. N. Engl. J. Med..

[11] Labelle M., Begum S., Hynes R.O. (2011). Direct signaling between platelets and cancer cells induces an epithelial-mesenchymal-like transition and promotes metastasis. Cancer Cell.

[12] Rachidi S., Metelli A., Riesenberg B., Wu B.X., Nelson M.H., Wallace C., Paulos C.M., Rubinstein M.P., Garrett-Mayer E., Hennig M. (2017). Platelets subvert T cell immunity against cancer via GARP-TGFβ axis. Sci. Immunol..

[13] Malehmir M., Pfister D., Gallage S., Szydlowska M., Inverso D., Kotsiliti E., Leone V., Peiseler M., Surewaard B.G.J., Rath D. (2019). Platelet GPIbα is a mediator and potential interventional target for NASH and subsequent liver cancer. Nat. Med..

[14] Nieswandt B., Hafner M., Echtenacher B., Männel D.N. (1999). Lysis of tumor cells by natural killer cells in mice is impeded by platelets. Cancer Res..

[15] Burn J., Sheth H., Elliott F., Reed L., Macrae F., Mecklin J.P., Möslein G., McRonald F.E., Bertario L., Evans D.G. (2020). Cancer prevention with aspirin in hereditary colorectal cancer (Lynch syndrome), 10-year follow-up and registry-based 20-year data in the CAPP2 study: A double-blind, randomised, placebo-controlled trial. Lancet.

[16] Simon T.G., Duberg A.S., Aleman S., Chung R.T., Chan A.T., Ludvigsson J.F. (2020). Association of Aspirin with Hepatocellular Carcinoma and Liver-Related Mortality. N. Engl. J. Med..

[17] Aster R.H. (1966). Pooling of platelets in the spleen: Role in the pathogenesis of “hypersplenic” thrombocytopenia. J. Clin. Investig..

[18] Ramadori P., Klag T., Malek N.P., Heikenwalder M. (2019). Platelets in chronic liver disease, from bench to bedside. JHEP Rep..

[19] Aryal B., Yamakuchi M., Shimizu T., Kadono J., Furoi A., Gejima K., Komokata T., Hashiguchi T., Imoto Y. (2018). Deciphering Platelet Kinetics in Diagnostic and Prognostic Evaluation of Hepatocellular Carcinoma. Can. J. Gastroenterol. Hepatol..

[20] Mazzaferro V., Regalia E., Doci R., Andreola S., Pulvirenti A., Bozzetti F., Montalto F., Ammatuna M., Morabito A., Gennari L. (1996). Liver transplantation for the treatment of small hepatocellular carcinomas in patients with cirrhosis. N. Engl. J. Med..

[21] Mazzaferro V., Llovet J.M., Miceli R., Bhoori S., Schiavo M., Mariani L., Camerini T., Roayaie S., Schwartz M.E., Grazi G.L. (2009). Predicting survival after liver transplantation in patients with hepatocellular carcinoma beyond the Milan criteria: A retrospective, exploratory analysis. Lancet Oncol..

[22] Child C.G., Turcotte J.G. (1964). Surgery and portal hypertension. Major Probl. Clin. Surg..

[23] Bruix J., Qin S., Merle P., Granito A., Huang Y.H., Bodoky G., Pracht M., Yokosuka O., Rosmorduc O., Breder V. (2017). Regorafenib for patients with hepatocellular carcinoma who progressed on sorafenib treatment (RESORCE): A randomised, double-blind, placebo-controlled, phase 3 trial. Lancet.

[24] Kudo M., Finn R.S., Qin S., Han K.H., Ikeda K., Piscaglia F., Baron A., Park J.W., Han G., Jassem J. (2018). Lenvatinib versus sorafenib in first-line treatment of patients with unresectable hepatocellular carcinoma: A randomised phase 3 non-inferiority trial. Lancet.

[25] Kamath P.S., Wiesner R.H., Malinchoc M., Kremers W., Therneau T.M., Kosberg C.L., D’Amico G., Dickson E.R., Kim W.R. (2001). A model to predict survival in patients with end-stage liver disease. Hepatology.

[26] Johnson P.J., Berhane S., Kagebayashi C., Satomura S., Teng M., Reeves H.L., O’Beirne J., Fox R., Skowronska A., Palmer D. (2015). Assessment of liver function in patients with hepatocellular carcinoma: A new evidence-based approach-the ALBI grade. J. Clin. Oncol..

[27] Bedossa P., Poynard T. (1996). An algorithm for the grading of activity in chronic hepatitis C. The METAVIR Cooperative Study Group. Hepatology.

[28] Choi A.Y., Chang K.J. (2022). Endoscopic Diagnosis of Portal Hypertension. Tech. Innov. Gastrointest. Endosc..

[29] R Core Team (2018). R: A Language and Environment for Statistical Computing. R Foundation for Statistical Computing, Vienna, Austria. https://www.R-project.org/.

[30] Therneau T. (2020). A Package for Survival Analysis in R. R Package Version 3.2-7. https://CRAN.R-project.org/package=survival.

[31] Mayo S.C., Shore A.D., Nathan H., Edil B.H., Hirose K., Anders R.A., Wolfgang C.L., Schulick R.D., Choti M.A., Pawlik T.M. (2011). Refining the definition of perioperative mortality following hepatectomy using death within 90 days as the standard criterion. HPB (Oxford).

[32] Pang Q., Qu K., Zhang J.Y., Song S.D., Liu S.S., Tai M.H., Liu H.C., Liu C. (2015). The Prognostic Value of Platelet Count in Patients With Hepatocellular Carcinoma: A Systematic Review and Meta-Analysis. Medicine (Baltimore).

[33] Hwang S.J., Luo J.C., Li C.P., Chu C.W., Wu J.C., Lai C.R., Chiang J.H., Chau G.Y., Lui W.Y., Lee C.C. (2004). Thrombocytosis: A paraneoplastic syndrome in patients with hepatocellular carcinoma. World J. Gastroenterol..

[34] Maithel S.K., Kneuertz P.J., Kooby D.A., Scoggins C.R., Weber S.M., Martin R.C., McMasters K.M., Cho C.S., Winslow E.R., Wood W.C. (2011). Importance of low preoperative platelet count in selecting patients for resection of hepatocellular carcinoma: A multi-institutional analysis. J. Am. Coll. Surg..

[35] Golriz M., Ghamarnejad O., Khajeh E., Sabagh M., Mieth M., Hoffmann K., Ulrich A., Hackert T., Weiss K.H., Schirmacher P. (2018). Preoperative Thrombocytopenia May Predict Poor Surgical Outcome after Extended Hepatectomy. Can. J. Gastroenterol. Hepatol..

[36] Scheiner B., Kirstein M., Popp S., Hucke F., Bota S., Rohr-Udilova N., Reiberger T., Müller C., Trauner M., Peck-Radosavljevic M. (2019). Association of Platelet Count and Mean Platelet Volume with Overall Survival in Patients with Cirrhosis and Unresectable Hepatocellular Carcinoma. Liver Cancer.

[37] Franchis R., Bosch J., Garcia-Tsao G., Reiberger T., Ripoll C., Abraldes J., Albillos A., Baiges A., Bajaj J., Bañares R. (2021). Baveno Vii—Renewing Consensus in Portal Hypertension. J. Hepatol..

[38] Qamar A.A., Grace N.D., Groszmann R.J., Garcia-Tsao G., Bosch J., Burroughs A.K., Maurer R., Planas R., Escorsell A., Garcia-Pagan J.C. (2008). Platelet count is not a predictor of the presence or development of gastroesophageal varices in cirrhosis. Hepatology.

[39] Berzigotti A., Seijo S., Arena U., Abraldes J.G., Vizzutti F., García-Pagán J.C., Pinzani M., Bosch J. (2013). Elastography, spleen size, and platelet count identify portal hypertension in patients with compensated cirrhosis. Gastroenterology.

